# B-cell precursor acute lymphoblastic leukemia and stromal cells communicate through Galectin-3

**DOI:** 10.18632/oncotarget.3409

**Published:** 2015-03-30

**Authors:** Fei Fei, Eun Ji Joo, Somayeh S. Tarighat, Isabelle Schiffer, Helicia Paz, Muller Fabbri, Hisham Abdel-Azim, John Groffen, Nora Heisterkamp

**Affiliations:** ^1^ Section of Molecular Carcinogenesis, Division of Hematology/Oncology and Bone Marrow Transplant, The Saban Research Institute of Children's Hospital Los Angeles, Los Angeles, CA, USA; ^2^ Division of Hematology/Oncology and Bone Marrow Transplant, Children's Hospital Los Angeles, Los Angeles, CA, USA; ^3^ Department of Pediatrics, Molecular Microbiology and Immunology, Keck School of Medicine, Norris Comprehensive Cancer Center, Children's Center for Cancer and Blood Diseases, Children's Hospital Los Angeles, University of Southern California, Los Angeles, CA, USA; ^4^ Leukemia and Lymphoma Program, Norris Comprehensive Cancer Center, Los Angeles, CA, USA; ^5^ Departments of Pediatrics and Pathology, Keck School of Medicine, University of Southern California, Los Angeles, CA, USA

**Keywords:** Lgals3, stroma, drug resistance, exosomes, microenvironment

## Abstract

The molecular interactions between B-cell precursor acute lymphoblastic leukemia (pre-B ALL) cells and stromal cells in the bone marrow that provide microenvironmentally-mediated protection against therapeutic drugs are not well-defined. Galectin-3 (Lgals3) is a multifunctional galactose-binding lectin with reported location in the nucleus, cytoplasm and extracellular space in different cell types. We previously reported that ALL cells co-cultured with stroma contain high levels of Galectin-3. We here establish that, in contrast to more mature B-lineage cancers, Galectin-3 detected in and on the ALL cells originates from stromal cells, which express it on their surface, secrete it as soluble protein and also in exosomes. Soluble and stromal-bound Galectin-3 is internalized by ALL cells, transported to the nucleus and stimulates transcription of endogenous *LGALS3* mRNA. When human and mouse ALL cells develop tolerance to different drugs while in contact with protective stromal cells, Galectin-3 protein levels are consistently increased. This correlates with induction of Galectin-3 transcription in the ALL cells. Thus Galectin-3 sourced from stroma becomes supplemented by endogenous Galectin-3 production in the pre-B ALL cells that are under continuous stress from drug treatment. Our data suggest that stromal Galectin-3 may protect ALL cells through auto-induction of Galectin-3 mRNA and tonic NFκB pathway activation. Since endogenously synthesized Galectin-3 protects pre-B ALL cells against drug treatment, we identify Galectin-3 as one possible target to counteract the protective effects of stroma.

## INTRODUCTION

Galectins are lectins that specialize in the recognition of galactose-containing structures. Galectin-3 (Lgals3) is a 30 kDa lectin that has cross-linking and lattice-promoting activity, through combination of an N-terminal domain that allows oligomerization with a C-terminal carbohydrate binding domain [1]. Galectin-3 is found extracellularly but is also present in the cytoplasm, and in the nucleus. Its carbohydrate-binding ability appears to be critical for extracellular functions, whereas intracellular Galectin-3 has many effects and molecular interactions that are largely carbohydrate-independent [2]. Importantly, elevated Galectin-3 levels are associated with numerous types of cancer, and frequently correlate with poor outcome [3–5]. In addition, inflammatory processes often are associated with elevated Galectin-3. However, the cellular origin of, and the reasons for increased Galectin-3 levels are poorly understood.

We recently showed that B-cell precursor acute lymphoblastic leukemia (pre-B ALL) cells derived from *galectin3−/−* mice are more sensitive to drug treatment than wild type cells, and that overexpression of Galectin-3 by retroviral transduction protects pre-B ALL cells against drug treatment [6]. Pre-B ALL can be subdivided into different categories based on underlying genetic defects such as the presence of the Bcr/Abl oncoprotein characteristic of Ph-positive ALL. However all types of pre-B ALL develop by malignant transformation of B-lineage precursor cells that normally mature in a regulated fashion under control of the bone marrow microenvironment by association with stromal cells. Primary human pre-B ALL cells are still largely dependent on stroma, and in patients who have evidence of minimal residual disease after initial chemotherapy, these cells are localized to the bone marrow. We found that bone marrow plasma samples of pre-B ALL patients contain elevated Galectin-3 levels as measured by ELISA [6]. Taken together, these studies suggest that Galectin-3 in the microenvironment may promote survival of pre-B ALL cells but did not establish the cellular origin of Galectin-3. In the current study, we show that Galectin-3 protein levels are dynamically regulated and induced through a reciprocal communication between leukemia cells and protective stromal cells, and are further increased by chemotherapeutic drug treatment. Interestingly, both stromal cells and ALL cells generate exosomes, but Galectin-3 is only present in microvesicles originating from stromal cells.

## RESULTS

### Stromal cells provide Galectin-3 to pre-B ALL cells

**Figure 1:**
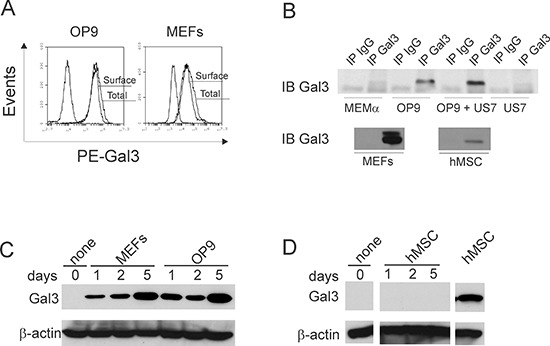
Protective stromal cells are the source of Galectin-3 present on ALL cells **A.** Flow cytometric analysis of surface and total Galectin-3 in OP9 and MEF stromal cells. Total Galectin-3 was measured in permeabilized cells. Non-marked plot, isotype control. **B.** Concentrated medium above cells examined by immunoprecipitation for the presence of Galectin-3. MEMα, MEMα + 20% FBS = complete medium without cells; hMSC, primary human mesenchymal stem cells. IPs on panels group analysis done together in single experiments, using equal cell numbers and culture times to condition medium. One of three independent experiments with similar results. **C, D.** Western blot analysis for Galectin-3 in human ALL BLQ1 cells plated on d0 on the cell types indicated above the lanes.

When co-cultured with stroma, pre-B ALL cells traffic dynamically between the stromal layer and the culture medium. Human pre-B ALL cells in direct contact with stroma contain Galectin-3 detectable by flow cytometry, but ALL cells harvested from the medium lack Galectin-3 [6]. To determine whether cellular contact of ALL cells with stroma induces Galectin-3 in ALL cells, we first performed flow cytometry to analyze Galectin-3 levels in stromal cells. As shown in Figure 1A, all cells within OP9 and mouse embryonic fibroblast (MEF) populations were positive for Galectin-3, with Galectin-3 mainly expressed on the cell surface (Figure 1A; OP9 MFI surface/total = 38900/51000; MEF MFI surface/total = 48000/51000).

Using immunoprecipitation, we also assayed the growth medium of murine and human stromal cells for secreted Galectin-3. Figure 1B shows that OP9 and MEFs secreted high amounts of this lectin, but human mesenchymal stem cells (hMSC; bottom panel), in comparison, secreted lower amounts. US7 ALL cells secreted no Galectin-3, compared to medium + FBS. However, there was approximately 1.5 fold more Galectin-3 in the culture supernatants of co-cultures of OP9 with human US7 ALL cells compared to OP9 cells alone, indicating that Galectin-3 secretion is stimulated by the interaction between these two cell types.

We next compared Galectin-3 protein levels in pre-B ALL cells harvested from co-cultures with different stromal cells. Western blot analysis confirmed that human BLQ1 ALL cells kept in suspension for 24 hours contain very low amounts of Galectin-3, and that this was significantly elevated when they were plated on MEF and OP9 stromal cells (Figure 1C). Similar results were obtained with TXL2 and US7 human ALL cells (not shown). Although hMSC did express Galectin-3, there was little Galectin-3 detectable in ALL cells that were plated on them (Figure 1D).

### Stromal exosomes but not ALL exosomes contain Galectin-3

Although the extracellular localization of Galectin-3 is well-established, secretion does not take place through the classical pathway, as Galectin-3 does not contain a signal sequence. To our knowledge, the exact mechanism through which it is exocytosed from stromal cells has not been described [7] [8]. Interestingly however, Mehul and Hughes [7] reported that murine macrophages produce vesicles containing Galectin-3. More recently, proteomic analysis on exosomes secreted by the bladder cancer cell line HT1376 showed these contain Galectin-3 [9]. This led us to consider the possibility that Galectin-3 may be transported through extracellular vesicles produced by stromal cells.

Galectin-3 is present in human serum and plasma, and is used or is under consideration as marker for different conditions ranging from cancer to acute heart failure [10]. To prevent possible contamination by Galectin-3 from FBS (see Figure 1B, Gal3 IP from MEMα+FBS), we used serum-free media to culture stromal OP9 cells, as well as human TXL2 ALL cells, for exosome isolation. Extracellular microvesicles secreted over a period of 48 hours into the culture supernatant were isolated, after a low-speed centrifugation of the supernatant to remove large cell debris and dead cells.

OP9 cells are not malignant and therefore growth factor-dependent. To assess the effect of growth factor deprivation due to FBS starvation, we compared apoptosis using FACS for Annexin V/PI in OP9 kept in serum-free medium for 24 and 48 hours. At 24 hours, OP9 cells were Annexin V negative (Suppl. Figure 1A), morphologically normal and adherent (not shown). At 48 hours, more OP9 cells had become Annexin V/PI-double positive but the human ALL cells essentially retained full viability even at 48 hours (Suppl. Figure 1B).

OP9 vesicles had an average size of 153 nm as measured by Nanosight (not shown), which is similar to sizes reported for murine bone marrow stromal cells, U87-MG glioblastoma cells, HT1080 lung sarcoma cells and glioblastoma multiforme (138–170, 196, 148 and 133 nm respectively) [11–13]. The OP9 microvesicles are also much smaller than the 1000–4000 nm size range reported for apoptotic bodies of endothelial cells [14].

**Figure 2:**
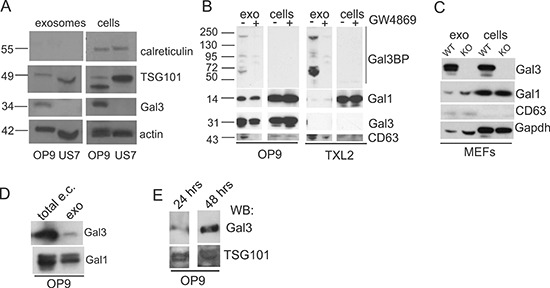
OP9 but not ALL cells generate Galectin-3 containing exosomes Western blot analysis on protein lysates of exosomes and the cells that secrete them. **A.** Expression of the ER marker calreticulin and the exosome marker TSG101 in exosomes and total cell lysates (cells) of OP9 stroma and US7 ALL. **B.** OP9 or TXL2 ALL cells treated for 48 hours with DMSO (−) or 20 μM exosome secretion inhibitor GW4869 (+). Exo, exosomes from the cells; cells, total lysates of the same cells. 20 μg protein/lane. **C.** Lysates of cells/exosomes of mouse embryonic fibroblasts (MEFs) from *lgals3+/+* (WT) or *lgals3 −/−* (KO) mice. 20 μg protein/lane. **D.** Total non-cell associated extracellular (e.c.) Galectin-3 present in 100 μl OP9 stromal-conditioned medium or exosome content equivalent to 100 μl medium. **E.** Exosomes isolated from OP9 cells held for 24 or 48 hours without FBS examined for Gal3 and TSG101 content using Western blotting.

Lysates from both OP9 and US7 microvesicle preparations were positive for the typical exosome marker TSG101 and were negative for calreticulin, an ER marker expressed on the surface of apoptotic cells and absent from exosomes [15, 16] (Figure 2A). Detection of the exosome marker CD63 [17] was decreased (Figure 2B) after treatment with the neutral sphingomyelinase inhibitor GW4869, which inhibits exosome production [18]. Based on these characteristics, we will refer to the microvesicles as exosomes.

OP9-derived exosomes contained substantial amounts of Galectin-3 compared to whole cell lysates, whereas US7 cells, and their exosomes, lacked Galectin-3 (Figure 2A). Similarly, exosomes of TXL2 ALL cells contained no detectable Galectin-3, and low levels of a related Galectin, Galectin-1, whereas OP9 stromal exosomes contained both Galectin-1 and Galectin-3-binding protein, Gal3BP (Figure 2B). Selectivity of incorporation was further confirmed by showing that exosome lysates of *lgals3−/−* MEFs contained Galectin-1, but not Galectin-3 (Figure 2C). The amount of Galectin-3 secreted by way of exosomes in OP9-conditioned medium was smaller than the total non-cell bound extracellular Galectin-3 measured in the medium (Figure 2D). We also compared Galectin-3 levels in exosomes secreted by OP9 stromal cells over a period of 24 and 48 hours into the culture supernatant. Both preparations were TSG101 positive and contained Galectin-3 (Figure 2E).

### Uptake of exosomes by ALL cells and stromal cells

**Figure 3:**
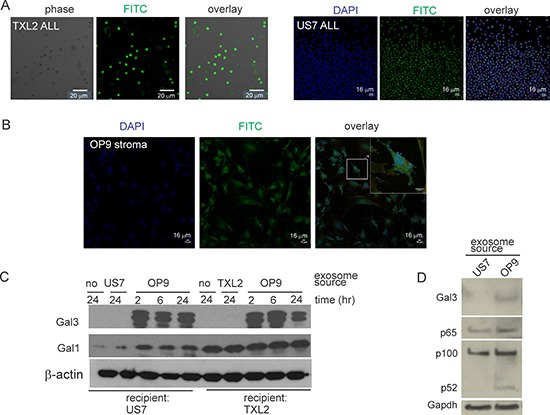
Uptake of OP9 exosome content by ALL cells **A, B.** Confocal microscopy of leukemia or stromal cells incubated with labeled heterologous exosomes. A: Human TXL2 ALL or US7 cells with OP9 exosomes. B: OP9 stromal cells with US7 exosomes. Bar, 20 μm (panel A-TXL2) or 16 μm (panel A-US7 and panel B). **C.** Western blots of lysates from US7 or TXL2 ALL cells cultured without OP9 stroma for 24 hours, then exposed to 50 μg/ml stromal or “self” ALL exosomes for 2–24 hours in serum free medium. 20 μg protein/lane. One of two independent experiments with similar results. **D.** Western blot of lysates (1.5 × 10^5^ cell equivalent/lane) from US7 cells cultured without OP9 or serum, then stimulated for 24 hours with 0.5 μg/μl US7 or OP9 exosomes. Antibodies used as indicated to the left.

To determine whether OP9 stromal exosomes can transport their content to ALL cells, we labeled OP9 exosomes with Exo-Green and exposed human US7 or TXL2 ALL cells to them for 2 hours. As shown in Figure 3A, confocal microscopy established that stromal exosome content was transferred to leukemia cells. Conversely, ALL exosomes were able to attach to OP9 stromal cells and deliver their content (Figure 3B).

To further investigate exosome-mediated Galectin-3 transport, we cultured human ALL cells without OP9 cells for 24 hours to remove stroma-produced Galectin-3 associated with the ALL cells. Western blotting confirmed loss of Galectin-3 but the cells retained endogenous Galectin-1 (Figure 3C, lanes labeled ‘no’ −24). We then exposed the cells to ALL or OP9 exosomes in the absence of serum. As shown in Figure 3C, in ALL cells treated with OP9 exosomes, Galectin-3 levels increased within 2 hours. Interestingly, Galectin-1 levels also increased in US7 cells. Higher levels of p100, p52 and p65 NFκB were also measured in US7 cells exposed for 24 hours to OP9 exosomes, compared the same cells exposed to US7 exosomes (Figure 3D). Thus, our experiments present evidence for a two-way communication exchange of materials between ALL cells and their stromal support cells via exosomes.

### Extracellular Galectin-3 is endocytosed and transported to the nucleus

**Figure 4:**
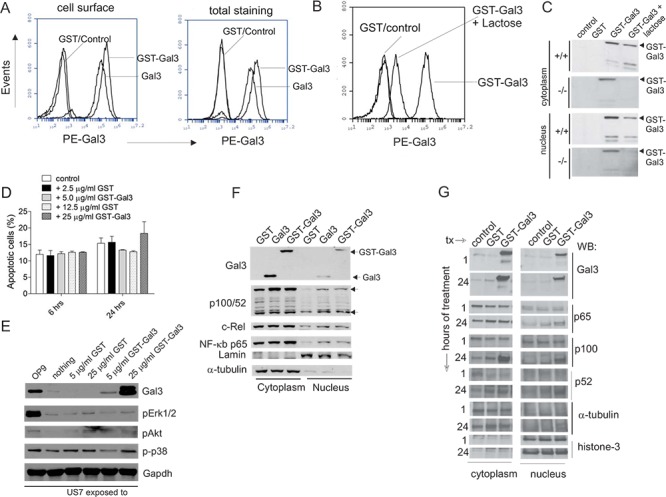
Exogenous Galectin-3 is internalized by human ALL cells **A.** Human TXL2 cells cultured without stroma for 24 hours were incubated for an additional 24 hours (control), with 12.5 μg/ml GST (GST), 12.5 μg/ml Galectin-3 or 25 μg/ml GST-Gal3. Flow cytometric analysis for cell surface or total (in permeabilized cells) Galectin-3 with anti-Galectin-3 antibodies as indicated. **B.** TXL2 cells cultured without OP9 cells for 24 hours, then treated with 12.5 μg/ml GST or 25 μg/ml GST-Galectin-3 plus 50 mM lactose for an additional 24 hours. Galectin-3 cell surface levels in TXL2 cells determined by flow cytometry. **C.** Western blot analysis of lysates from *wt* and *lgals3* −/− pre-B ALL cells exposed for 24 hours to nothing (control); 12.5 μg/ml GST, 25 μg/ml GST-Gal3 or 25 μg/ml GST-Gal3 + 50 mM lactose. The two smaller proteolytic products were also present in this input GST-Galectin-3 (26 kDa GST + 27.5 kDa Galectin-3) recombinant protein. **D.** Effect of exogenous Galectin-3 on apoptosis of US7 cells indicated as percentage Annexin V+, PI+ cells at the indicated time points determined by FACS. One of two experiments with similar results. **E.** Western blot analysis for signal transduction pathway activation in US7 cells cultured without OP9 for 24 hours, then treated for 24 hours as indicated above the panel. **F.** Nuclear and cytoplasmic lysates of TXL2 cells treated with GST, Galectin-3 or GST-Galectin-3 for 24 hours in the absence of OP9 cells analyzed by Western blot. **G.** Nuclear and cytoplasmic fractions of TXL2 cells kept without stroma for 24 hours, then stimulated with the proteins indicated above the lanes for 1 hour or 24 hours. Control, cells re-plated on OP9. Lamin, histone-3 and α-tubulin, loading controls for nuclear and cytoplasmic fractions, respectively.

Exosomes from stromal cells contain proteins and other macromolecules besides Galectin-3, including lectins such as Galectin-1 (Figure 2, 3), which also binds N-acetyl lactosamine-modified glycoproteins [1]. To identify the specific signal generated in the ALL cells by stimulation with extracellular Galectin-3, we first removed the stromal-produced Galectin-3 associated with the ALL cells. To this end, we cultured TXL2 cells without OP9 cells for 24 hours and confirmed Galectin-3 loss by flow cytometry (Figure 4A, samples labeled control). We then stimulated the ALL for 24 hours with recombinant human GST-Galectin-3 and with recombinant GST as additional control, in the continued absence of stroma. The N-terminal GST tag enhances but is not predicted to change the specificity of the natural lattice-forming ability of Galectin-3. However, we also included Galectin-3 without the GST tag as comparison. Interestingly, Galectin-3 was detected on the surface of ALL cells that had been exposed to either Galectin-3 or GST-Galectin-3, showing that exogenously added Galectin-3 stably binds to structures on the surface of ALL cells (Figure 4A). High-affinity carbohydrate ligands identified for Galectin-3 include cell surface proteins containing tri-and tetra antennary N-linked glycans that are N-acetyl lactosamine modified. Therefore, inhibition of Galectin-3 binding by competition with lactose, although it is a lower-affinity ligand, can be used to determine if interactions are partly carbohydrate-lectin dependent. Figure 4B shows that the binding of GST-Galectin-3 to ALL cells was reduced by lactose, demonstrating that docking of Galectin-3 on the ALL cell surface is mediated at least in part through binding to cell surface glycosylated molecules.

Many studies support the concept that the function of Galectin-3 depends on where it is localized. We therefore treated pre-B ALL cells grown without stroma with GST-Gal3 for 24 hours in the presence and absence of lactose and prepared cytoplasmic and nuclear fractions to determine if the exogenous Galectin-3 was endocytosed and transported to the nucleus, or if it was degraded. As shown in Figure 4C, part of the GST-Gal3 had become nuclear, and co-incubation with lactose decreased both cytoplasmic as well as nuclear levels. *Lgals3+/+* and *lgals3−/−* pre-B ALL cells gave similar results, indicating that endogenous Galectin-3 is not required for uptake or nuclear transport of extracellular Galectin-3.

Treatment of T-cells with extracellular Galectin-3 was reported to stimulate apoptosis (reviewed in [19]) so we asked if extracellular Gal3 treatment of pre-B ALL cells also induces death in this cell type. In contrast to the reported effects on T-cells, a 24 hour treatment with the highest concentration of GST-Gal3 (25 μg/ml) only marginally increased the percentage of apoptotic cells compared to controls (Figure 4D).

We next examined if stimulation with soluble Gal3 activates intracellular signal transduction pathways in pre-B ALL cells. However, stimulation with exogenous Galectin-3 for 24 hours did not result in increased activation of Erk1/2, p38 or Akt as compared to a positive control consisting of US7 cells stimulated by plating on OP9 stroma (Figure 4E).

We also evaluated NFκB pathway activation. We found that a 24-hour stimulation of cells with GST-Galectin-3 but not GST increased cytoplasmic p100 and nuclear p100/p50, c-Rel and RelA/p65 in TXL2 cells (Figure 4F). Although alternative NFκB pathway activation involves proteolytic processing of p100 to p52 with translocation of p52 to the nucleus, increased p100 levels and the migration of p100 to the nucleus have also been reported [20, 21]. We examined the kinetics of NFκB activation in more detail by comparing cytoplasmic and nuclear fractions of TXL2 cells stimulated for 1 hour and for 24 hours with GST-Galectin-3. As shown in Figure 4G, GST-Galectin-3 was recovered both in the nucleus and cytoplasm at the 1 hour time point, indicating rapid endocytosis and transport. Increased cytoplasmic and nuclear p100, and nuclear p65 increases in GST-Galectin-3 stimulated cells were evident only after 24 hours, consistent with the typical kinetics of NF-κB activation [22].

### Auto-activation of Gal3 transcription

**Figure 5:**
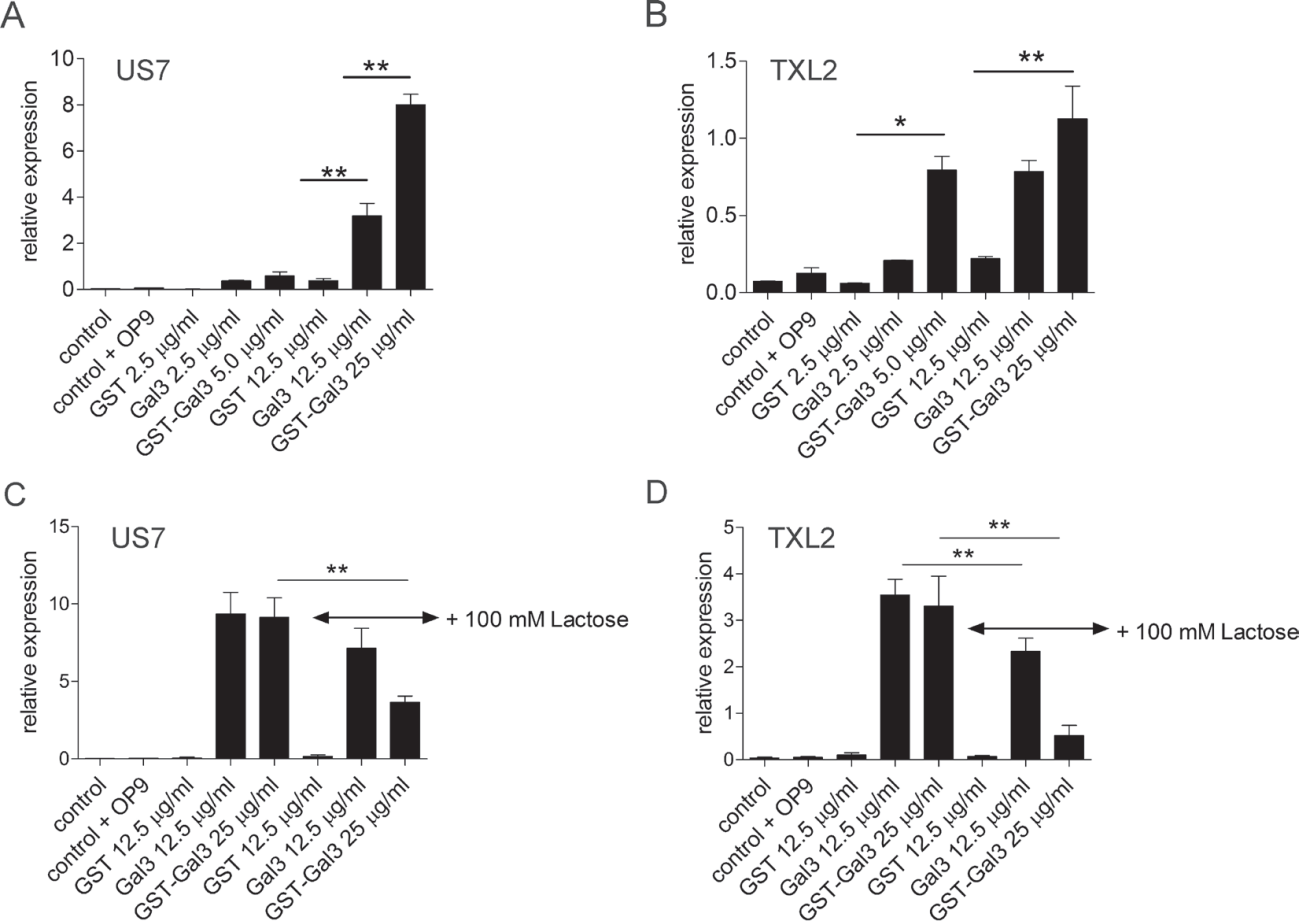
Stimulation by extracellular Galectin-3 induces transcription of endogenous Galectin-3 in pre-B ALL cells TXL2 or US7 cells held without stroma for 24 hours to remove Galectin-3 were treated with the indicated recombinant proteins for 24 hours, or with nothing (control) in the continued absence of stroma. Samples designated ‘control+OP9’ are untreated ALL cells re-plated on OP9 stroma for 24 hours. *LGALS3* mRNA levels on US7 and TXL2 were measured by qRT-PCR and are expressed as relative to (percentage of) those of the reference gene *GAPDH* (*p < 0.05; **p < 0.01).

These results raise the question if stimulation of ALL cells with extracellular Galectin-3 modulates transcription. Since ALL + OP9 co-cultures have higher Galectin-3 protein levels than OP9 cells alone, we considered the possibility that Galectin-3 may be able to stimulate its own transcription. To test this hypothesis, we removed ALL cells from OP9 stromal support for 24 hours, then stimulated them with exogenous Galectin-3. RNA levels of human *LGALS3* in the ALL cells were quantified by real-time RT/PCR using human-specific primers. As shown in Figure 5, ALL cells in suspension (control samples) contained very low *LGALS3* mRNA levels. Cycle threshold values for control US7 and TXL2 (Figure 5C, D) were 30.3 ± 0.2 and 28.6 ± 0.4, respectively. However, compared to stimulation with a molar equivalent of control GST protein, Galectin-3 and GST-Galectin-3 proteins highly stimulated the transcription of endogenous *LGALS3* mRNA in both US7 and TXL2 pre-B ALL cells (Figure 5A, B). To address to which degree Galectin-3-stimulated transcription is dependent on binding of Galectin-3 to cell surface glycoproteins, we also performed this experiment in the presence of competing, extracellularly added lactose. As shown in Figure 5C and 5D, the inclusion of lactose reduced, but did not eliminate the induction of *LGALS3* mRNA in both ALLs. This result is consistent with the incomplete inhibition by lactose of GST-Galectin-3 cell surface binding (Figure 4B) and internalization (Figure 4C), and suggests that Galectin-3 may have less affinity for lactose compared to the natural carbohydrate ligands on the cell surface. Alternatively, some of the Galectin-3 binding, internalization and nuclear transport may be carbohydrate-independent.

### Long-term drug treatment increases Galectin-3 in ALL cells in contact with stroma

Increased Galectin-3 protein levels are frequently associated with inflammation. Since we previously found that the development of environmentally-mediated drug resistance (EMDR) in ALL-stromal co-culture models correlates with a profile of increased expression of genes typically associated with inflammation [23], we investigated the effect of chemotherapy on Galectin-3 levels in co-cultures of stromal and leukemia cells. The irradiation of OP9 cells causes minimal changes in overall gene expression or Galectin-3 protein levels [24]. Also, Galectin-3 levels in the medium of irradiated OP9 cells that had been treated for 6 days with vincristine were identical to those secreted by control cells not treated with vincristine (Suppl. Figure 2A), and there were no changes induced by a 6-day nilotinib treatment in MEFs or OP9 cells (Suppl. Figure 2B, C).

**Figure 6:**
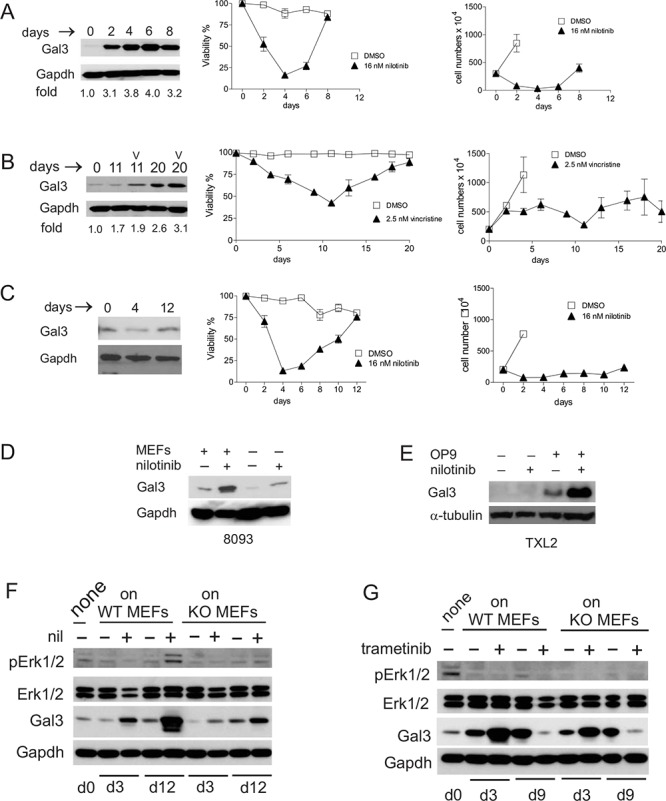
Chemotherapeutic drug treatment induces endogenous Galectin-3 in pre-B ALL cells when in contact with stromal cells **A.** Left panel, Western blot analysis for Galectin-3 expression; middle panel, viability and right panel, cell counts of murine Bcr/Abl-positive 8093 pro/pre-B ALL cells treated with nilotinib. Western blot: fold increase is with respect to Galectin-3 levels on d0. Cell counts: cell numbers are not indicated for all days in the control samples since removal of cells was necessitated by overcrowding due to exponential cell growth. **B.** Human US7 ALL cells treated with 2.5 nM vincristine (v) in the presence of irradiated OP9 cells. Panels as in A. **C.** Murine 8093 ALL cells treated with 16 nM nilotinib but without direct contact with MEFs. Panels as in A. **D.** 8093 cells treated with or without 16 nM nilotinib for 48 hours in the presence or absence of MEFs. **E.** Human Ph-positive TXL2 cells treated with or without 1 μM nilotinib for 72 hours in the presence or absence of OP9 cells. **F, G.** 8093 pro/pre-B ALL cells cultured without MEFs for 24 hours (input d0 samples designated ‘none’), then plated on *gal3+/+* [WT] or *−/−* [KO] MEFs as indicated above the panels, were harvested on different days of treatment with 16 nM nilotinib (F) or 10 nM trametinib (G) Antibodies used for Western blots are shown to the left in each panel. Gapdh, loading control. F, representative image, one of two independent experiments. In all experiments, fresh drug was added with each fresh medium change.

When stromal-dependent mouse 8093 pro/pre-B ALL cells are co-cultured with MEFs and treated with nilotinib, the viability of the leukemia cells decreases and their proliferation is abolished (Figure 6 right panels d0–4). As reported previously [23], upon prolonged treatment, and in continued presence of stromal support, such cells regain the ability to proliferate and developed tolerance to the drug (Figure 6A, middle and right panels d4–8). As shown in Figure 6A (left panel), when the ALL cells were newly plated on MEFs they initially contained low levels of Galectin-3 protein. After the ALL cells had been in co-culture with MEFs for 6 days, while simultaneously undergoing nilotinib treatment, Galectin-3 levels increased approximately 4-fold in them compared to d0.

Human US7 cells developed tolerance to 2.5 nM vincristine after 2–3 weeks of continued treatment when provided with OP9 stromal support, as measured by increased viability from d12 onwards (Figure 6B mid panel), although vincristine continued to be cytostatic as evidenced by the lower cell numbers (Figure 6B right panel). Galectin-3 protein levels also increased in the ALL cells during this period (Figure 6B left panel).

To determine if cell contact between stroma and ALL cells was required for the observed drug-induced increase in Galectin-3 protein, we measured Galectin-3 protein levels in ALL cells separated from the stromal cells by a Transwell membrane during drug treatment. Interestingly, the levels of Galectin-3 did not increase under these conditions (Figure 6C). To demonstrate that drug treatment increased Galectin-3 levels above those induced by the presence of stroma, we performed a side-by side comparison. As shown in Figure 6D, nilotinib treatment or contact with MEFs increased Galectin-3 in ALL cells, and treatment with nilotinib in the presence of stromal support resulted in the highest expression. Similar results were obtained with human Ph-positive TXL2 cells treated for 72 hours with nilotinib, in the presence or absence of OP9 cells (Figure 6E).

To further demonstrate that ALL cells under stromal cell support can produce endogenous Galectin-3 protein when stressed by drug therapy, we treated mouse 8093 pre-B ALL cells with nilotinib over a period of 14 days while in co-culture with *lgals3−/−* and +/+ MEFs. In non-drug treated cells (samples designated —), Galectin-3 protein was not greatly increased compared to levels present in the input cells on d0. However, nilotinib treatment highly induced Galectin-3 on d3 and d12 of drug treatment, and part of this was produced endogenously in the ALL cells since it was also measured in cells grown with Galectin-3 −/− MEFs (Figure 6F).

We previously reported that the emergence of environmentally mediated drug resistance (EMDR) correlates with increased levels of pErk1/2 in mouse 8093 ALL cells on d11 of nilotinib treatment [23]. We found similar results on d12 with 8093 cells co-cultured with MEFs lacking Galectin-3, although pErk1/2 levels were lower than in 8093 cells plated on +/+ MEFs (Figure 6F, compare d12 + samples). We conclude that stromal-dependent pre-B ALL cells can be induced to produce endogenous Galectin-3 protein when they are stressed by nilotinib or vincristine drug treatment.

To determine if Erk activation is involved in Galectin-3 induction in ALL cells under drug treatment we treated ALL cells with the Mek inhibitor trametinib. Trametinib was able to reduce Erk activation seen in long-term drug-treated cells on d9 and interestingly, endogenous ALL Galectin-3 protein levels on d9 were also suppressed (Figure 6G).

### Induction of endogenous Galectin-3 mRNA in ALL cells stressed by drug treatment

**Figure 7:**
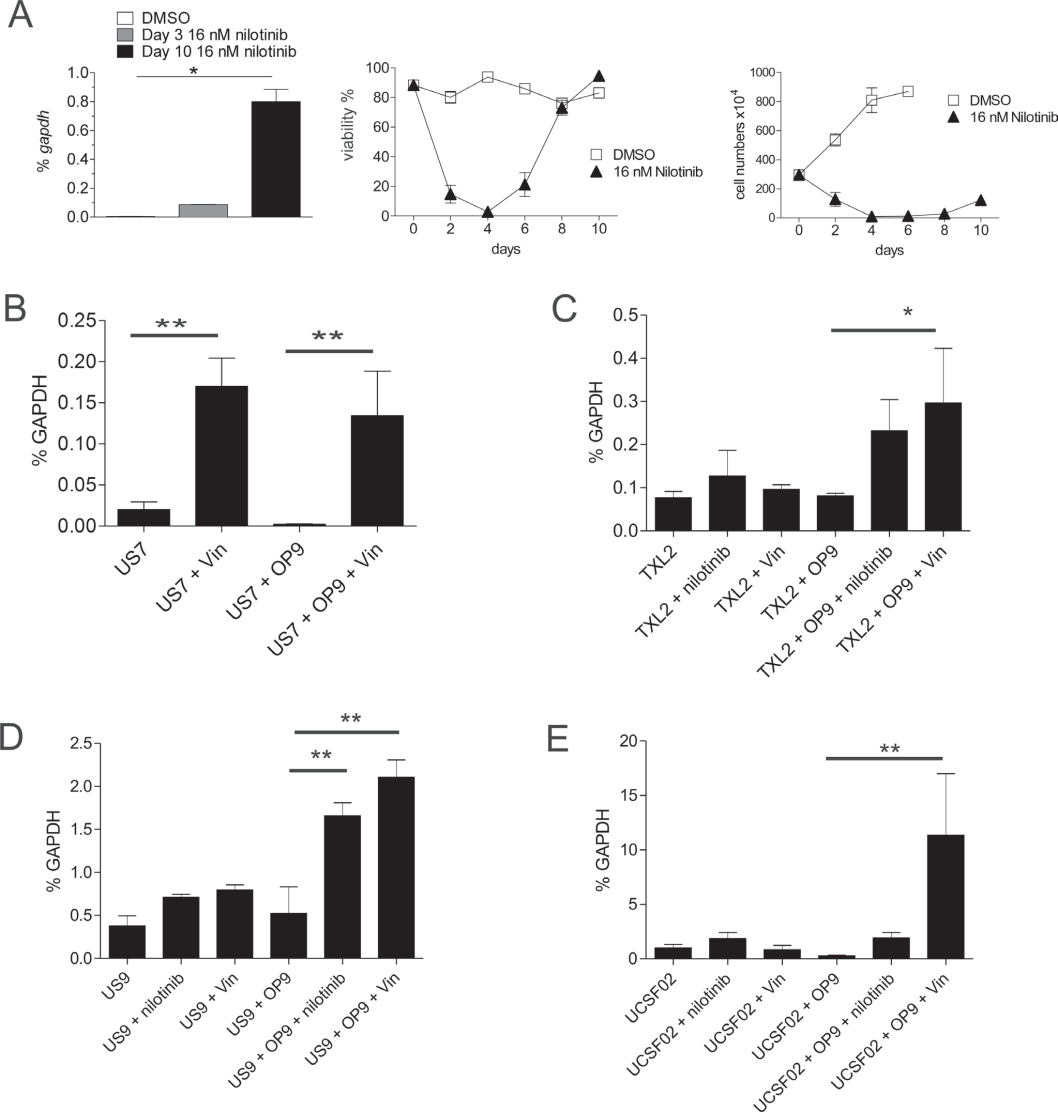
Transcription of *Lgals3* is activated by drug treatment **A.** Real-time RT/PCR for *lgals3* mRNA in 8093 Bcr/Abl-expressing pre-B ALL cells treated with nilotinib in the presence of irradiated MEFs on the indicated days. Left, qRT-PCR; mid and right, viability and cell numbers of the samples. One of four independent experiments with similar results. **B–E.** *LGALS3* mRNA measured by qRT-PCR as percentage of the reference gene *GAPDH* in B: human US7; C: human TXL2; D: human US9; and E: human UCSFO2 ALL cells treated as indicated for 24 hours with 5 nM vincristine or 1 μM nilotinib. US7 and TXL2, one of two experiments with similar results.

We complemented these experiments by investigating *Lgals3* mRNA levels in drug-treated mouse and human cells. *Lgals3* was highly increased in mouse ALL cells on d10 of nilotinib treatment, when the culture resumed proliferation (Figure 7A). *LGALS3* mRNA levels in human US7 ALL cells treated with vincristine for 24 hours were significantly elevated compared to vehicle-treated cells (Figure 7B). Higher expression was also found in these cells on d5 and d12/16 of treatment (not shown). Human TXL2 in co-culture with OP9 stroma, treated for 24 hours with nilotinib or with vincristine, also exhibited significantly elevated mRNA levels (Figure 7C). Similar results were obtained using different Ph-positive patient-derived human ALL cells including US9 treated with vincristine or nilotinib, and UCSF02 ALL cells treated for 24 hours with vincristine or nilotinib (Figure 7D, 7E). We conclude that induction of *LGALS3* is a common characteristic of mouse and human ALL cells when they are treated with chemotherapy while in direct contact with stromal cells.

### Inhibition of canonical NFκB signaling in pre-B ALL cells

Since stimulation of pre-B ALL cells with soluble Galectin-3 activates NFκB, and also induces Galectin-3 transcription, we investigated if canonical NFκB inhibition would affect Galectin-3 expression. We used BMS345541 [25], an inhibitor which has been reported to affect CLL, AML and T-ALL [26–28] but with unknown effects on pre-B ALL.

**Figure 8:**
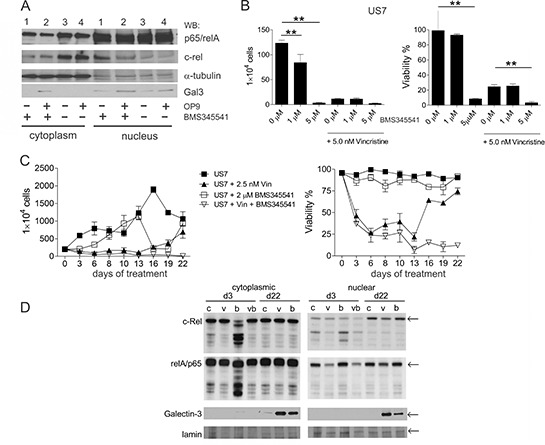
Inhibition of canonical NFκB signaling **A.** Cytoplasmic or nuclear lysates (10 μg/lane) from TXL2 cells cultured for 24 hours without OP9 stroma to remove preexisting Galectin-3, then treated for 24 hours with 10 μM BMS345543 (lanes 1 and 2) or DMSO (lanes 3 and 4) in the absence (lanes 1 and 3) or presence (lanes 2 and 4) of OP9 stromal support as also indicated below the panel. TXL2 ALL cells were harvested from the medium. **B.** US7 cells treated with the indicated concentrations of BMS345541 alone or combined with 5 nM vincristine. Cell numbers (left panel) and viability (right panel) were determined after 72 hours using Trypan blue exclusion. Results shown are representative of two independent experiments with both US7 and TXL2 with very similar results (**p < 0.001). **C.** long-term treatment of US7 cells in the presence of irradiated stromal cells with 2.5 nM vincristine, 2 μM BMS345541, or both as indicated. Fresh drug was added with every medium change. **D.** Western blot analysis of samples from (C) taken at days 3 and 22 as indicated. v, vincristine; bv, BMS345541 + vincristine; b, BMS345541; c, control DMSO. Not enough cells remained on d22 for Western blot analysis of the bv treated culture.

We exposed TXL2 cells for 24 hours to 10 μM drug with and without the presence of OP9 cells, and harvested the ALL cells from the medium (Figure 8A). Since these ALL cells were not in direct contact with OP9 cells, they contained very low levels of Galectin-3, consistent with results in Figure 6C. Interestingly, some Galectin-3 was detectable in the nucleus of the ALL cells, indicating that soluble OP9-secreted Galectin-3 is also transported to the nucleus. Short-term inhibition of the canonical NFκB pathway had no effect on Galectin-3 nuclear transport or levels. Reduction of total levels of c-Rel and p65 was detectable within 24 hours of treatment (Suppl. Figure 3A).

We further measured proliferation and viability after 72 hours of BMS345541 treatment. BMS345541 was cytostatic at 1 μM for US7 (Figure 8B left panel) and TXL2 cells (Suppl. Figure 3B left panel) even when they were grown with stromal support. At 5 μM it also was cytotoxic, reducing the viability of both ALLs (Figure 8B and Suppl. Figure 3B right panels) over the course of 3 days of treatment. In combination with 500 nM nilotinib (AMN107), BMS345541 further suppressed the proliferation and viability of TXL2 cells (Suppl. Figure 3B). BMS345541 also eradicated mouse 8093 ALL cells co-cultured with stroma at 5 μM concentrations (not shown).

Assays for drug resistance promoted by stromal protection require the use of moderate doses of drugs that do not immediately eradicate the leukemia cells, and EMDR typically evolves over a period of many days in co-culture with protective stroma. For example, treatment with 2.5 nM vincristine as single agent over a 28-day period allowed the detection of drug-tolerant cells (Suppl. Figure 3C). However, 5 μM BMS345541 killed the cells within 12 days (Suppl. Figure 3C). Therefore we selected 2 μM BMS345541 as a lower dose for longer-term treatment that is still able to reduce nuclear c-Rel and p65 levels (Suppl. Figure 3D, 3E). As shown in Figure 8C, monotreatment with 2 μM BMS345541 did not affect viability of the ALL cells and only was cytostatic during the first 6 days of treatment, showing that drug-tolerance against this inhibitor also developed rapidly. Western blot analysis showed that c-Rel and p65 were affected on d3 of treatment, but on d22 levels were indistinguishable from that of controls (Figure 8D d22 compare lanes ‘c’ and ‘b’). In the d22 cultures treated only with vincristine or BMS345541, Galectin-3 levels in the cytoplasm and in the nucleus were elevated compared to controls (Figure 8D d22 lanes ‘v’ and ‘b’). Interestingly, in the cultures treated simultaneously with vincristine and BMS345551, drug tolerant cells did not emerge (Figure 8C, vincristine + BMS345541 treated samples) and all cells died.

## DISCUSSION

Subsequent to its identification in 1989 as Mac-2, a galactose-binding lectin and macrophage marker [29], Galectin-3 has been implicated in a wide spectrum of pathological conditions including cancer and inflammation. Studies on Galectin-3 are complicated by the finding that it is endogenously synthesized in some cells, also exported by others, and can be moreover found present in the nucleus and/or cytoplasm [30]. We previously reported that bone marrow plasma samples of pre-B ALL patients contain elevated Galectin-3. Here, we have carefully analyzed the origins of Galectin-3 relevant to this type of leukemia. Surprisingly, B-cell precursor ALL cells that depend on stromal support for their growth and survival were found to synthesize little or no endogenous Galectin-3. This in marked contrast to a more mature B-lineage cancer, diffuse large B-cell lymphoma, which was reported to contain high levels of endogenous Galectin-3 mRNA [31]. Similarly, we did not detect any Galectin-3 in exosomes produced by pre-B ALL cells. Thus some more mature B-cell cancers differ from B cell precursor leukemia in that the former make their own Galectin-3.

Pre-B ALL cells in the bone marrow are in close proximity to stromal cells and are thought to make physical contact via structures that, in one study [32], were named stromal synapses in analogy to the immunological synapse formed between antigen-presenting B-cells and T-cells [32]. We found that stromal fibroblasts are a major source of extracellular Galectin-3. These cells express a high percentage of the total Galectin-3 on their surface, and we found soluble and microvesicle-packaged Galectin-3 in the culture supernatant of stromal OP9 cells. We can not exclude the possibility that Galectin-3 in the bone marrow is initially secreted in microvesicles that release their content over time, as this was reported by Mehul and Hughes for macrophage-produced vesicles [7]. However we argue that soluble Galectin-3 is biologically significant, since we found elevated soluble Galectin-3 in the bone marrow plasma of pre-B ALL patients using an ELISA [6]. Moreover, although ELISA-based assays for soluble Galectin-3 are widely used for detection in human peripheral blood serum/plasma, proteomics on human plasma exosomes [33] report the presence of Lgals3BP, but not Galectin-3. Thus it is unlikely that Galectin-3 is an abundant component of human peripheral blood exosomes. In agreement with this, in exosomes from serum of control and ALL patient peripheral blood (TSG101-positive microvesicles with an average size distribution of 92 ± 2.6 nm and 98 ± 0.4 nm respectively on Nanosight), Galectin-3 was not readily detectable (results not shown). Thus it will be more of interest to investigate if bone marrow of pre-B ALL patients with minimal residual disease have different levels of extracellular Galectin-3 compared to those who have no detectable residual ALL cells.

The question arises how fibroblasts present Galectin-3 to pre-B ALL cells. We found that close cell-cell contact is important for efficient transfer of high levels of stromal Galectin-3 to ALL cells, since ALL cells that are not attached to stroma lost significant amounts of Galectin-3 over a period of hours. This suggests that Galectin-3 is presented to ALL cells in a concentrated form through proximity to stromal fibroblasts, bound to the fibroblast-secreted extracellular matrix, to the surface of fibroblasts themselves, or concentrated in packages of exosomes. Exosomes may be a vehicle of choice for some types of cell-cell communication in which a ‘synapse’ is formed that allows very precise local delivery of such vesicles. Indeed, exosome-mediated communication was recently shown to be an important component of the immunological synapse between T-cells and antigen-presenting B-cells [34]. Chen et al previously placed Galectin-3 at the immunological synapse, where it negatively regulates TCR-mediated T-cell activation, and identified Alix, which is involved in exosome biogenesis [35] as a Galectin-3 binding protein [36]. Since exosomes only need to transverse a short distance between cells and are prevented from dissipation because of the confining boundary of the synapse [34], this is an attractive model for stromal-produced Galectin-3 uptake by pre-B ALL cells.

Alternatively, a recent study demonstrated that exposure of cells to soluble recombinant Galectin-3 triggers the formation of clathrin-independent carriers (CLICs) in the plasma membrane, promoting endocytosis of specific cargo proteins such as CD44 and β1 integrin [37]. Our studies showed that recombinant soluble Galectin-3 (Figure 4C, 4F, 4G) as well as non-recombinant OP9-stromal secreted Galectin-3 (Figure 8A) are transported to the nucleus. Although the fate of CLICs has not been tracked, Erbb3 may be transported to the nucleus of prostate cancer cells through such a mechanism [38] and we speculate that Galectin-3 and select cargo/client proteins may make this journey together. Surprisingly, we recovered Galectin-3 in the nuclear fraction of pre-B ALL cells within one hour of its addition, suggesting that this is a mechanism that allows the ALL cells to rapidly respond to Galectin-3 produced by the microenvironment.

Exposure of some cell types to extracellular Galectin-3 results in programmed cell death. Treatment of CD4 CD8 double-negative T-cells, T-cell leukemia, diffuse large B cell lymphoma and colon cancer cell lines for 4–48 hours with 300–15000 nM Galectin-3 was reported to result in apoptosis of the recipient cells [39–42]. However we found that treatment of pre-B ALL cells with 25 μg/ml GST-Gal3 or 12.5 μg/ml Gal3 (corresponding to 463 nM Galectin-3) for 24 hours did not promote apoptosis. We note that serum Galectin-3 concentrations reported in cancer patients are up to 32 nM [43–45], in line with our data on Galectin-3 levels in bone marrow plasma from ALL patients [6]. Although we can not exclude the possibility that Galectin-3 becomes concentrated on the surface of cells, resulting in much higher effective levels than 32 nM, our results do not support the concept that the Galectin-3 produced by stroma has either a pro-apoptotic (Figure 4D), or strong anti-apoptotic (not shown) function. In concordance with this, we were unable to detect changes in levels of Akt or Erk1/2 activation in Galectin-3-stimulated cells.

Between 6 and 24 hours after stimulation of the pre-B ALL cells with Galectin-3, we did detect activation of NFκB. Also, stimulation of pre-B ALL cells with extracellular Galectin-3 increased the levels of Galectin-3 mRNA in those cells. Since NFκB transcription factors contribute to regulation of Galectin-3 expression in other cell types ([46]; [47] and references therein) Galectin-3 transcription may be directly activated by NFκB. However, because Galectin-3 protein also migrates to the nucleus, where it can regulate pre-mRNA splicing [30], it is possible that regulation of Galectin-3 mRNA levels and NFκB activation are indirectly linked.

We found a surprisingly strong induction of endogenous Galectin-3 mRNA and protein expression in the pre-B ALL cells when they were in contact with the stromal cells while subjected to the stress of cytotoxic drug treatment. The *de novo* induction occurred over the course of days, correlated with Erk activation and the emergence of drug tolerance. Cells in suspension did not induce Galectin-3 protein. Since we previously showed that increasing levels of endogenous Galectin-3 protein by overexpression from a retroviral vector protects pre-B ALL cells against drug treatment [6], we propose a model in which the induction of endogenous Galectin-3 protein production requires cell-to-cell contacts between stromal and ALL cells and is aided by chemotherapy (Suppl. Figure 4). We note in this context that in human patients, massive cell death due to chemotherapy may generate inflammation in the bone marrow and could increase levels of extracellular Galectin-3 and protective intracellular Galectin-3 in pre-B ALL cells though an auto-induction mechanism.

Although inhibitors specific for Galectin-3 have not yet been reported, our results suggest that Erk pathway inhibition with trametinib, and canonical NFκB pathway inhibition with BMS345541, reduce but do not eliminate drug-induction production of Galectin-3 protein in ALL cell co-cultures with stroma. Erk inhibitors (manuscript in preparation) and BMS345541 also reduced pre-B ALL cell proliferation when the ALL cells were co-cultured with stroma and this effect could be partly through inhibition of stromal Galectin-3 uptake by ALL cells and/or its endogenous production.

BMS345541 cytotoxicity is unlikely to be mediated only through Galectin-3. A short-term treatment with varying drug concentrations produced a stochastic effect on pre-B ALL viability, with cytotoxicity at concentrations of 5 μM or higher, but with mainly a cytostatic effect at concentrations of 2 μM when the cells were co-cultured with stroma. This suggests that this compound may have off-target effects in which the drug also inhibits other targets at higher concentrations. However, the combination of a relatively low BMS345541 dose with vincristine was able to overcome EMDR, suggesting that canonical NFκB pathway inhibition, and/or BMS345541 in particular, could be tested in combination with other drugs for treatment of minimal residual disease of pre-B ALL in the bone marrow.

Communication between pre-B ALL cells and the stroma takes place through multiple contacts including, among others, SDF1α with CXCR4, and α4β1 integrins with VCAM-1 or fibronectin [48, 49]. Thus Galectin-3 is not the only mode of communication between these cell types and our results using MEFs lacking Galectin-3 suggest that extracellular Galectin-3 is not absolutely required for long-term drug tolerance of mouse pre-B ALL cells (not shown). However, since MEFs also produce Galectin-1, which is highly related, may have overlapping functions with Galectin-3, and is moreover constitutively expressed in ALL cells, the possibility that lack of extracellular Galectin-3 is compensated for by the presence of Galectin-1 can not be excluded. Further studies will be needed to determine to which extent the functions of these two lectins overlap.

## MATERIALS AND METHODS

### Cells and culture

OP9 stromal cells (CRL-2749) were from the American Type Culture Collection (ATCC). Human mesenchymal stem cells (hMSC) were obtained from TUCGT (Tulane University Center for Gene Therapy). Human pre-B ALLs, mouse 8093 Bcr/Abl P190-expressing transgenic ALL and mouse *gal3−/−* and +/+ pre-B ALL cells have been previously described [49–53]. Galectin-3 null mutant mice on a C57Bl/6J background (stock 006338) were purchased from The Jackson Laboratories (Bar Harbor). Sex and age-matched C57Bl/6J mice were used as source of WT cells. MEFs were generated from E14.5 embryos as described previously [54]. Human leukemia cells and OP9 cells were co-cultured in MEM-α medium supplemented with 20% FBS, 1% L-glutamine and 1% penicillin/streptomycin. In all experiments, stromal cells were mitotically inactivated by irradiation. OP9 stroma exosomes were purified from non-irradiated OP9 cells.

### Drugs

The Bcr/Abl tyrosine kinase inhibitor nilotinib (AMN107) was obtained from Novartis (Basel, Switzerland) and was used to treat Ph-positive (Bcr/Abl–expressing) mouse and human ALLs. Nilotinib was dissolved in DMSO and stored at −20°C. Vincristine sulfate obtained as a solution from Hospira Worldwide Inc. (Lake Forest, IL, USA) was used to treat Ph-negative ALLs. BMS345541 was purchased from Sigma-Aldrich (St Louis, MO, USA) and Trametinib was obtained from Cayman Chemical (Ann Arbor, Michigan, USA).

### Galectin expression

Extracellular and total Galectin-3 levels by flow cytometry were measured on an Accuri C6 flow cytometer (BD Biosciences, USA). To measure total Galectin-3 using FACS, cells were first fixed and permeabilized with permeabilization buffer (eBioscience, San Diego, USA) before staining with PE-Galectin-3 antibodies. To investigate Galectin-3 secretion, irradiated MEFs, OP9, OP9 plus 3 × 10^6^ US7 cells or hMSC were plated in a 6-well plate at around 3 × 10^5^ cells/well. After 4 days, medium (3 ml) was collected and concentrated to around 600 μl by centrifugation through Amicon centrifugal filter units (Millipore, Bedford, MA) with a 10 K molecular weight cut-off. 250 μl was used for immunoprecipitation using IgG or Galectin-3 antibodies.

Real-time RT-PCR was performed as described previously [55] on RNA samples in triplicate. The following primers were used for mouse *galectin-3* (*lgals3;* NM_001145953.1): TCAGGTGAGCGGCACAGAGA (Fw) and TCCAGAGCCAGCTAAGGCATCGT (Rv) to give an 80-bp fragment. Human *LGALS3* (NM_002306.3) primers for real time RT/PCR were: GGCCACTGATTGTGCCTTAT (Fw) and TCTTTCTTCCCTTCCCCAGT (Rv) to give a 224-bp fragment.

To measure mRNA levels during drug exposure, US7, UCSFO2, US9 or TXL2 cells were held in MEM-α medium with 20% FBS, 1% L-glutamine and 1% penicillin/streptomycin for 24 hours without OP9 stroma, then treated or not with 5 nM vincristine or 1 μM nilotinib for an additional 24 hours. Parallel samples were kept on OP9 for 24 hours, and then treated for 24 hours, or not, with vincristine or nilotinib. To measure the effect of exogenous Galectin-3 stimulation, human ALL cells harvested from the medium of co-cultures with OP9 were kept for 24 hours in medium without OP9 stroma. Cells were then exposed to medium, or molar equivalent GST, GST-Galectin-3 or Galectin-3 for an additional 24 hours or plated on OP9 as positive control. Viability of US7 and TXL2 cells kept without OP9 for 24 hours was >90%.

### Exosomes

Cells were cultured in MEM-α medium with 20% FBS, 1% L-glutamine and 1% penicillin/streptomycin, then kept for 48 hours in serum-free medium. Supernatants were centrifuged 30 minutes at 1500 g to remove debris and an Amicon 10 k filter was used to concentrate the medium. Exosomes were isolated using either an ExoQuick-TC kit (System Biosciences, Mountain View, CA, USA) or an Exo-spin kit (Cell Guidance Systems LLC, Carlsbad CA) according to the manufacturer's instructions from OP9, TXL2 and US7 culture supernatants. Quality of a representative OP9 exosome isolate was determined by Nanoparticle Tracking analysis using a Nanosight instrument (Malvern Instruments). Mean particle size distribution was 153 +/− 3.3 nm with a concentration of 14.11 +/− 1.14 E8 particles/ml. In some experiments, cells were incubated with DMSO or 20 μM GW4869 (Cayman Chemical, Ann Arbor, MI, USA) for 48 hours. Exosomes were labeled using an Exo-Glow Exosome labeling kit (System Biosciences, Mountain View, CA, USA) according to the manufacturer's instructions. Exo-Green labeled OP9 exosomes were incubated for 2 hours with TXL2 cells in serum-free medium. TXL2 cells were seeded one day earlier at 1 × 10^6^ cells/well of a 24-well plate. After extensive washing with PBS to remove non-attached exosomes, a 5-minute incubation with CellMask Deep Red (Life Technologies, Grand Island NY) as plasma membrane stain was followed by fixing, permeabilization and DAPI stain.

TXL2 or US7 cells were first cultured without OP9 stromal support for 24 hours, followed by treatment with 50 μg/ml exosomes isolated from OP9, US7 or TXL2 cultures for different time points in serum-free medium as indicated in Figure 3C. For detection of NFκB pathway activation, we stimulated 0.2 × 10^6^ 24-hr serum-starved US7 cells without stroma in a 200 μl volume with 100 μg OP9 exosomes for 24 hours. ALL cells were extensively washed, then lysed in 40 μl 2xSDS-SB.

### GST/GST-Galectin-3 constructs

GST-Galectin-3 was constructed using human Galectin-3 and has been previously described [56]. GST and GST-Galectin 3 were further concentrated using a Centricon-10 (Amicon) filter and purified using an endotoxin-free kit (Norgen Biotech, Canada). The GST moiety was removed from the fusion protein by overnight incubation with thrombin.

### Western blotting and antibodies

Cells were lysed in SDS lysis buffer (100 mM NaCl, 500 mM Tris, pH 8.0, 10% SDS). For detection of NF-κB p100/52, p65 and c-Rel, a nuclear extraction kit (Thermo Scientific, MA, USA) was used to separate nuclear and cytoplasmic fractions. Cell extracts were subjected to 8–15% sodium dodecyl sulfate-polyacrylamide gel electrophoresis. Antibodies for Western blotting include: Galectin-3 (cat. 125402) (Biolegend, San Diego, USA), Galectin-1 (cat. Ab25138) and TSG101 (cat. ab30871; detects murine and human proteins) (Abcam, Cambridge, MA, USA), CD63, Erk1/2, NF-κB p65 (sc-8008 Figure 4G) (Santa Cruz Biotechnology, USA), phospho-Erk1/2, NF-κB p65 (cat. 8242) and calreticulin (cat. 2891S; detects murine and human proteins) (Cell Signaling Technology, USA), NF-κB p100/52 (cat. 06–413) (Millipore, USA). Gapdh (Chemicon International, USA or Millipore MAB374), α-tubulin (Oncogene Science, Cambridge, USA), lamin A, Histone H3 (Cell Signaling Technology, USA) or β-actin (cat. GTX109639, GeneTex) were used as a loading control.

### Statistical analysis

Statistical analysis was performed with Prism software. Data are presented as mean ± SD of triplicate samples. Statistical significance of differences between groups was evaluated using one-way-ANOVA or paired *t*-test. The value of *p* < 0.05 was considered to be statistically significant.

## SUPPLEMENTARY FIGURES



## Abbreviations

ALL: acute lymphoblastic leukemia
BM: bone marrow
ELISA: enzyme-linked immune assay
EMDR: environmental-mediated drug resistance
Lgals3: Galectin-3
MEF: mouse embryonic fibroblast
MFI: mean fluorescence intensity
PB: peripheral blood
Ph: Philadelphia
