# Supplementary material for: B-cell precursor acute lymphoblastic leukemia and stromal cells communicate through Galectin-3
Source: Oncotarget. 2015 Mar 30;6(13):11378–94. doi: 10.18632/oncotarget.3409 (PMC4484463; doi:10.18632/oncotarget.3409)
Supplement: Supplementary file 1 [file oncotarget-06-11378-s001.pdf]

SUPPLEMENTARY FIGURES

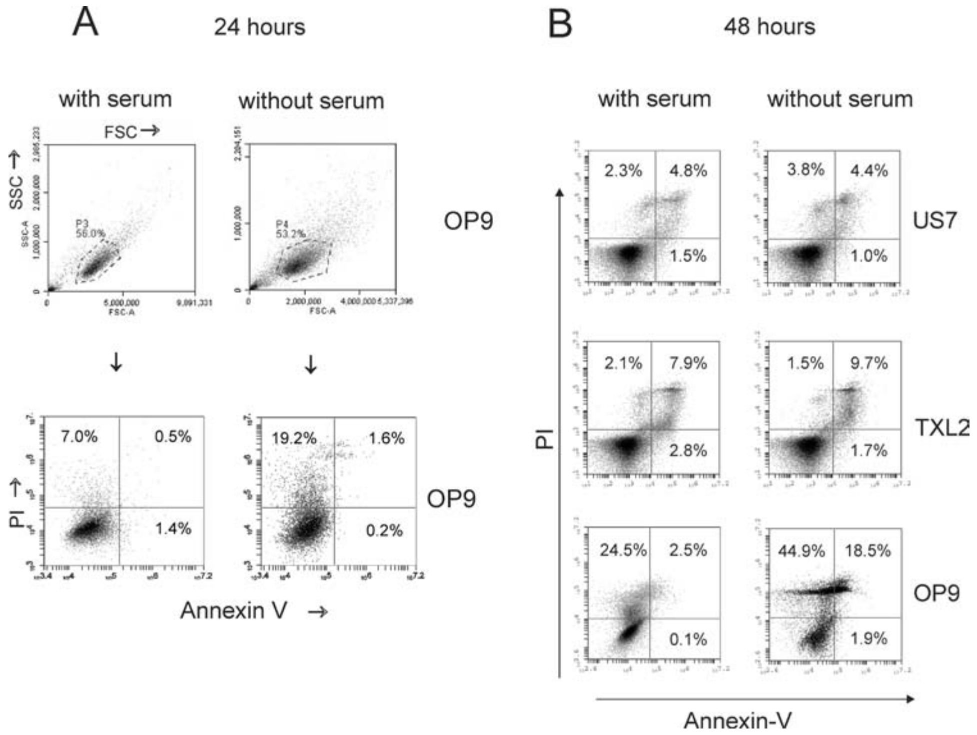

Supplementary Figure 1: Effect of serum starvation on OP9 stromal and pre-B ALL viability. FACS analysis for apoptosis using Annexin V and propidium iodide (AnnexinV FITC Apoptosis Detection Kit II, BD Pharmingen, San Jose, CA) on **A.** OP9 cells cultured for 24 hours with and without serum or **B.** US7, TXL2 and OP9 cells cultured alone, for 48 hours with or without serum. Note that even with serum, a substantial percentage of OP9 cells was Annexin V-negative but PI-single positive (7% at 24 hrs; 17.1 to 24.5% at 48 hrs).

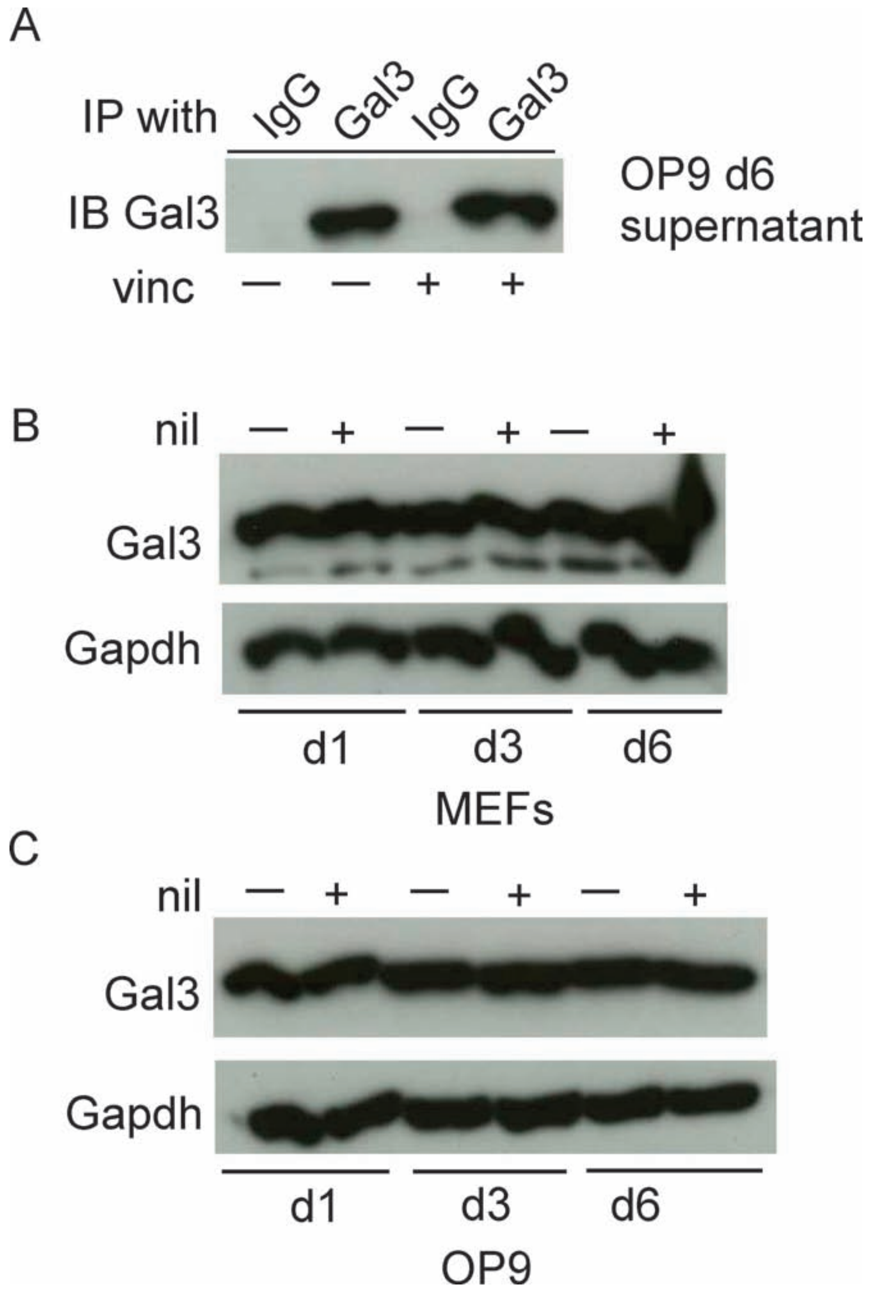

Supplementary Figure 2: Nilotinib or vincristine treatment does not affect levels of Galectin-3 in stromal cells. A. Medium above irradiated OP9 cells treated for 6 days as indicated was assayed for Galectin-3 by immunoprecipitation. **(B, C)**, Lysates of MEFs **B.** or OP9 cells **C.** treated with nilotinib for the indicated number of days assayed for Galectin-3 by Western blot.

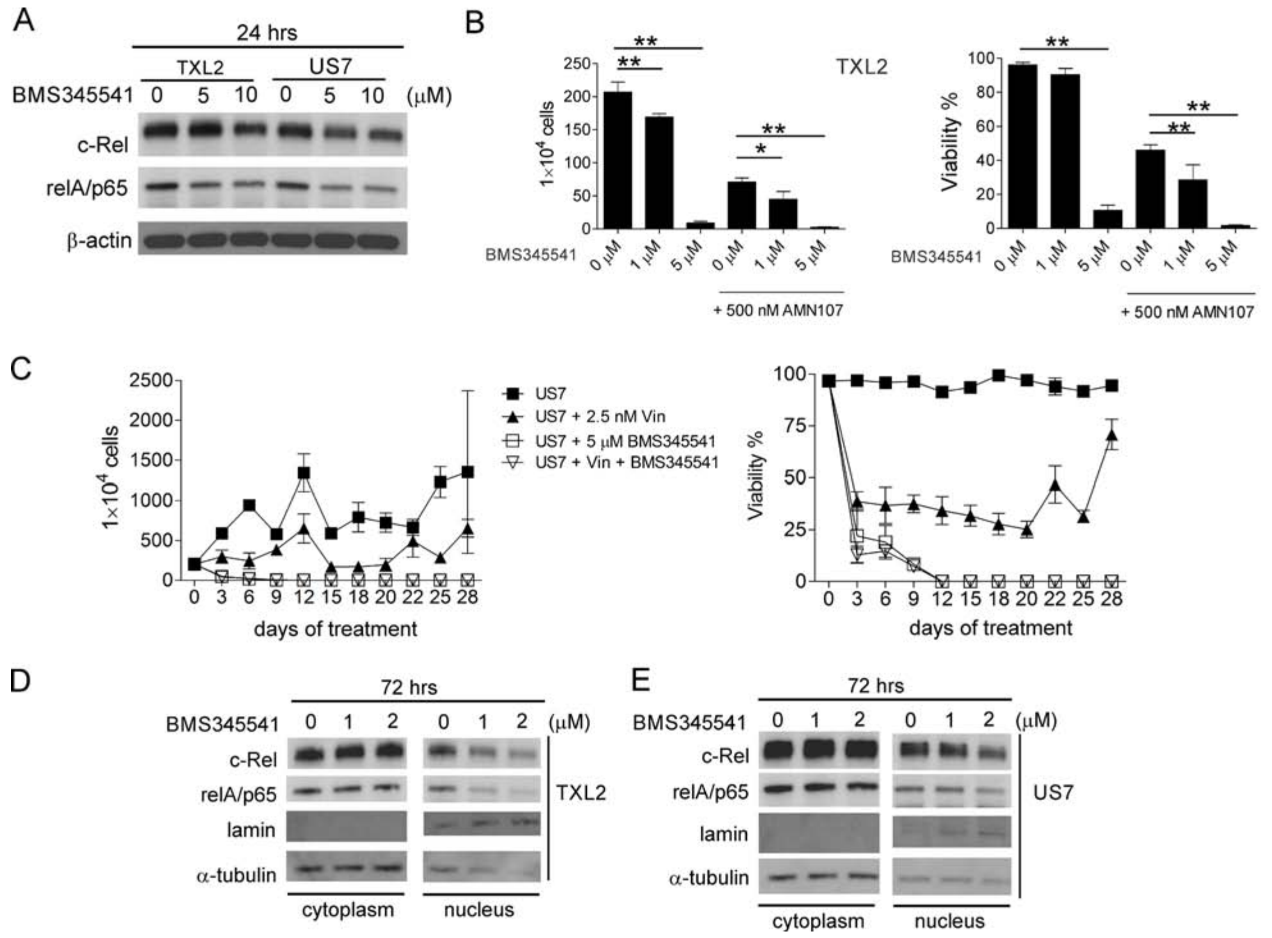

Supplementary Figure 3: Determination of non-lethal BMS345541 mono-treatment dose for EMDR of pre-B ALL cells in the presence of stroma. A. Western blot analysis with the indicated antibodies on 20 μg total cell lysates of US7 or TXL2 cells treated for 24 hours with 0, 5 and 10 μM BMS345541. **B.** Cell counts and viability of TXL2 cells grown on OP9 stroma and treated for 72 hours with the indicated drugs (**p* < 0.05, ***p* < 0.001). **C.** Long-term viability and cell counts of US7 cells over a 28-day period when treated with solvent, or the indicated drugs. Fresh drugs were added with every medium change. **(D, E)** Cytoplasmic and nuclear extracts (10 μg protein/lane) of 72-hour treated TXL2 **D.** or US7 **E.** cells using 0, 1 or 2 μM BMS345541. All cells were grown with irradiated OP9 stromal cells.

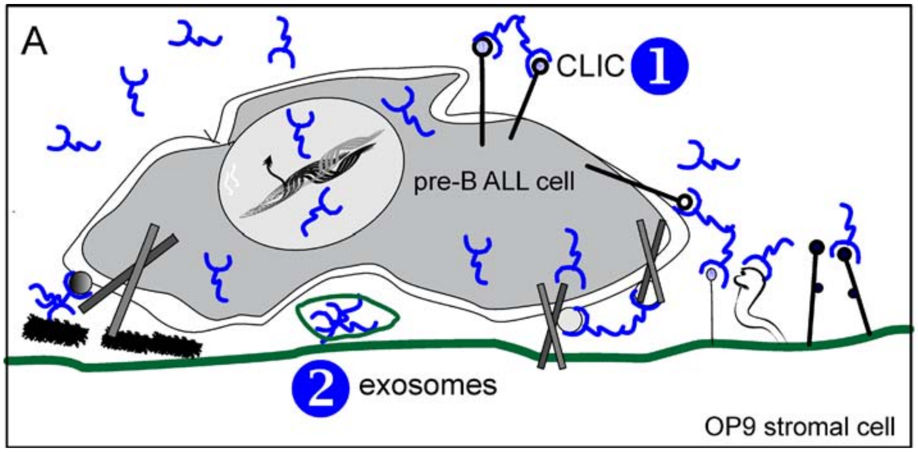

Supplementary Figure 4: Summary model for pre-B ALL protection by Galectin-3. A. Steady state survival and proliferation. We here show that bone marrow fibroblasts but not pre-B ALL cells synthesize Galectin-3. Galectin-3 is expressed on the surface of the fibroblasts and is also secreted by them. ALL cells are known to form multiple contacts with such stromal cells and their secreted extracellular matrix, which could be strengthened by extracellular Galectin-3 lattice-promoting/crosslinking activities. **(1)** Soluble Galectin-3 binds to glycoproteins on the surface of pre-B ALL cells and may clusters cargo into endocytic pits that could be internalized as clathrin-independent carriers (CLIC) [37]. Cargo for Galectin-3 in pre-B ALL cells has not been determined but could include β1 integrin, CD44 and CD45 [37, 40]. **(2)** We show here that stromal cell-produced exosomes carrying Galectin-3, Galectin-1, and Galectin-3 binding protein form a second route of transport. Exosomes may be concentrated in fibroblastic synapses that could include the pre-B receptor and integrins that have been found in other studies [32]. Our data show that soluble Galectin-3 is spontaneously internalized by pre-B ALL cells, and persists for at least 24 hours in the cytoplasm and nucleus. Furthermore, transport to the nucleus is rapid and occurs within 1 hour. We found that exposure to soluble Galectin-3 activates NFκB and induces increased Galectin-3 mRNA transcription. Since experiments with BMS345541 show that inhibition of the canonical NFκB pathway even in the presence of stromal support is cytostatic and cytotoxic for pre-B ALL cells, stromal Galectin-3 may protect ALL cells through auto-induction of Galectin-3 mRNA and tonic NFκB pathway activation.

(*Continued*)

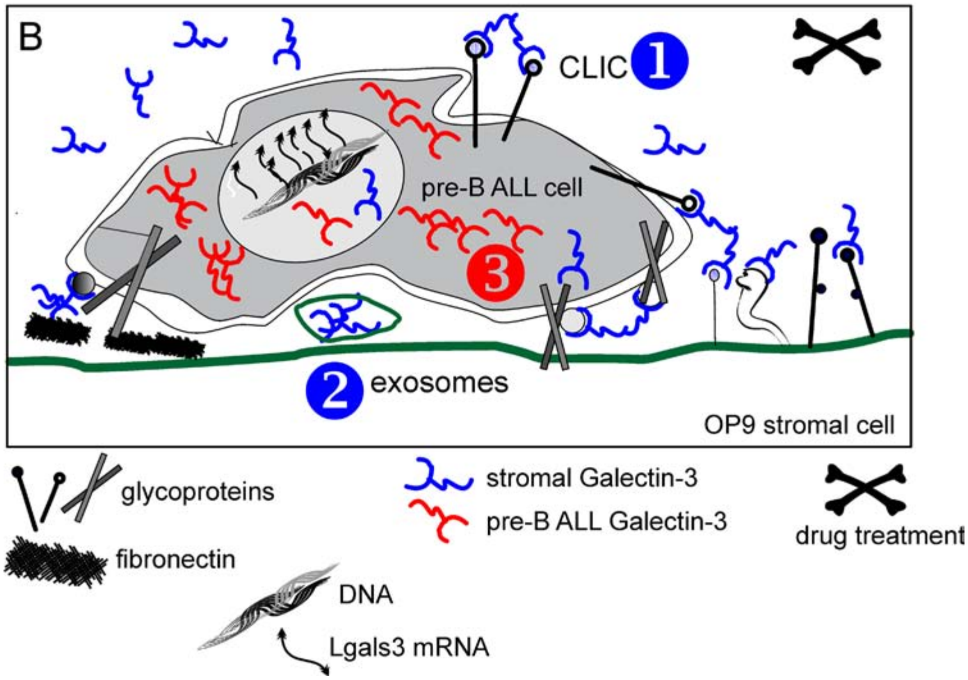

Supplementary Figure 4 (*Continued*): B. Environmentally-mediated drug resistance. Our current and previous results show that treatment of pre-B ALL cells with vincristine or nilotinib while in co-culture with stroma causes death of the majority of leukemia cells within a period of days. **(3)** Survivor drug-treated ALL cells in physical contact with stroma induce transcription of Galectin-3 mRNA, and increase levels of Galectin-3 protein (this study). Our previous studies showed that increased endogenously produced Galectin-3 protein enhances, and loss of endogenous Galectin-3 protein decreases ALL cell viability when these cells are treated with drugs [6]. Thus this is a protective mechanism. Prolonged culture in the presence of stroma under continued drug treatment results in the appearance of ALL cells with restored or enhanced levels of tyrosine phosphorylation (in Bcr/Abl-positive leukemias), pErk1/2 [23, 52], nuclear c-Rel and RelA/p65 (this study). **(1) (2)** Part of the increased Galectin-3 originates endogenously in the pre-B ALL cells but our results are consistent with stromal-produced Galectin-3 also contributing to the high level Galectin-3 expression through its continued cross-linking/lattice-promoting activities on the cell surface of the ALL cells, its auto-stimulatory activity on endogenous Galectin-3 mRNA production in the nucleus of the ALL cells, and NFκB pathway stimulation.
